# Vascular wall imaging in reversible cerebral vasoconstriction syndrome – a 3-T contrast-enhanced MRI study

**DOI:** 10.1186/s10194-018-0906-7

**Published:** 2018-08-30

**Authors:** Chun-Yu Chen, Shih-Pin Chen, Jong-Ling Fuh, Jiing-Feng Lirng, Feng-Chi Chang, Yen-Feng Wang, Shuu-Jiun Wang

**Affiliations:** 1 Department of Neurology, Neurological Institute, Taipei Veterans General Hospital, Taipei, 112 Taiwan; 2 Faculty of Medicine, National Yang-Ming University School of Medicine, Taipei, Taiwan; 3 Institute of Clinical Medicine, National Yang-Ming University, Taipei, Taiwan; 4 Division of Translational Research, Department of Medical Research, Taipei Veterans General Hospital, Taipei, Taiwan; 5 Department of Radiology, Taipei Veterans General Hospital, Taipei, Taiwan; 6 Brain Research Center, National Yang-Ming University, Taipei, Taiwan

**Keywords:** Reversible cerebral vasoconstriction syndromes, Thunderclap headache, Vascular wall imaging, Contrast enhancement

## Abstract

**Background:**

Limited histopathology studies have suggested that reversible cerebral vasoconstriction syndromes (RCVS) does not present with vascular wall inflammation. Previous vascular imaging studies have had inconsistent vascular wall enhancement findings in RCVS patients. The aim of this study was to determine whether absence of arterial wall pathology on imaging is a universal finding in patients with RCVS.

**Methods:**

We recruited patients with RCVS from Taipei Veterans General Hospital prospectively from 2010 to 2012, with follow-up until 2017 (*n* = 48). We analyzed the characteristics of vascular wall enhancement in these patients without comparisons to a control group. All participants received vascular wall imaging by contrasted T1 fluid-attenuated inversion recovery with a 3-T magnetic resonance machine. The vascular wall enhancement was rated as marked, mild or absent.

**Results:**

Of 48 patients with RCVS, 22 (45.8%) had vascular wall enhancement (5 marked and 17 mild). Demographics, clinical profiles, and cerebral artery flow velocities were similar across patients with versus without vascular wall enhancement, except that patients with vascular wall enhancement had fewer headache attacks than those without (*p* = 0.04). Follow-up imaging completed in 14 patients (median interval, 7 months) showed reduced enhancement in 9 patients, but persistent enhancement in 5.

**Conclusion:**

Almost half of our RCVS patients exhibited imaging enhancement of diseased vessels, and it was persistent for approximately a third of those patients with follow-up imaging. Both acute and persistent vascular wall enhancement may be unhelpful for differentiating RCVS from central nervous system vasculitis or subclinical atherosclerosis.

## Background

Reversible cerebral vasoconstriction syndrome (RCVS) is a unifying term for a variety of clinical-radiological syndromes characterized by recurrent thunderclap headaches and reversible multifocal cerebral vasoconstrictions [1–4]. RCVS is not uncommon and potentially devastating because it is associated with a high risk of complications, such as posterior reversible encephalopathy syndrome, ischemic stroke, intracerebral hemorrhage and cortical subarachnoid hemorrhage (SAH) [3, 5–10]. The diagnosis is based primarily on angiography demonstrating cerebral vasoconstrictions and their reversibility, but its differentiation from central nervous system (CNS) vasculitis can be challenging [11, 12].

Conventional arterial imaging, such as computed tomography or magnetic resonance angiography (MRA), can be used to evaluate vascular stenosis in RCVS. However, the specificity of such imaging is limited by similar luminal defects being the result of other pathologies [13]. The small caliber and tortuosity of intracranial vessels hamper visualization of vascular walls by conventional imaging techniques [13]. After being used initially to characterize the luminal stenosis in carotid atherosclerotic disease [14], black-blood imaging techniques have been applied to intracranial vascular wall visualization and characterization of vascular wall pathologies, including intracranial atherosclerosis [15], vasculitis [16], arterial dissection [17], aneurysm [18] and RCVS [19, 20].

It is not known whether there are pathological vascular wall changes underlying RCVS vasoconstrictions. Generally, the limited histopathological data available do not support the presence of arterial wall inflammation in patients with RCVS [12, 21, 22]. However, in one case report, marked vascular wall enhancement was noted in a patient with cocaine vasculitis [23], and cocaine use has been considered to be an important etiology of RCVS [9, 12, 24]. In a recent case series, 3 patients with RCVS showed no apparent vascular wall enhancement on contrasted T1 fluid-attenuated inversion recovery (FLAIR) imaging, whereas marked vascular wall enhancement was found in 3 patients with CNS vasculitis and 1 patient with cocaine vasculopathy [19]. In another case series, 4 of 13 patients with RCVS had mild enhancement on T1-weighted sequences with fat suppression and a saturation band [20]; the remaining 9 patients had no enhancement. No congruous conclusions can be drawn from these studies. Therefore, we aimed to determine whether absence of arterial wall pathology on imaging is a universal finding in patients with RCVS or could be characteristic of a subgroup of RCVS patients, as well as to further refine these clinical-pathological syndromes into more specific disease entities.

## Methods

### Study subjects

We recruited 62 patients presenting with acute severe headaches prospectively from the headache clinic and emergency department at Taipei Veterans General Hospital from March 2010 to September 2012. Each subject completed a detailed headache intake form and provided comprehensive medical and headache histories before undergoing clinical and neurological examinations. Brain magnetic resonance imaging (MRI), MR venography and MRA were performed to exclude intracranial lesions attributable to the patients’ headache. Spinal tap with cerebrospinal fluid analysis was performed to support diagnosis if patients agreed. Subjects were hospitalized to expedite completion of these diagnostic investigations if conditions allowed.

Diagnosis of RCVS required fulfillment of the following criteria: (1) at least two acute-onset severe headaches (thunderclap headaches), with or without focal neurological deficits; (2) vasoconstrictions demonstrated on MRA; and (3) reversibility of vasoconstrictions demonstrated by at least one follow-up MRA within 3 months. The diagnostic criteria were based on the definition of “benign (or reversible) angiopathy of the central nervous system” proposed by the International Classification of Headache Disorders, second edition (ICHD-2) (Code 6. 7.3) [25] and the essential diagnostic elements of RCVS proposed by Calabrese et al. [1]. The criteria were also in concordance with the newly proposed criteria for “headaches attributed to RCVS” in the ICHD-3 beta version (code 6.7.3) [26]. The exclusion criteria included: RCVS due to secondary causes, SAH or other intracranial disorders (but cortical SAH was allowed), and subjects with a poor vascular wall imaging quality, due to either a failure to focus on the large proximal vessels or difficulty with interpretation due to obscuration by motion artifacts.

### Vascular wall imaging

All subjects underwent sequential brain MRI examinations with adequate sequences to exclude intracranial lesions, using a previously reported procedure [6, 7] except that a 3-T MR machine was used (MR750®, GE Medical Systems, Milwaukee, WI). Sequential MRAs were performed in all subjects until their vasoconstrictions normalized or until 3 months after disease onset.

We employed a vascular wall imaging protocol adapted from that proposed by Swartz et al. [15]. In brief, the protocol consisted of T1-weighted black blood vessel wall sequence (single inversion recovery-prepared two-dimensional fast spin echo acquisition with a 22 × 22 cm^2^ field of view, 512 × 512 acquired matrix, 1.5 mm slice thickness, total slab thickness of 2–3 cm, and repetition/inversion/echo times of 2263/860/13 ms) before and after intravenous gadolinium administration (with constant scan parameters). All sequences were monitored for quality to ensure appropriate orientation to capture affected arteries at sites of stenosis. The acquisitions were targeted to ensure sampling of the middle cerebral arteries (MCAs).

**Fig. 1:**
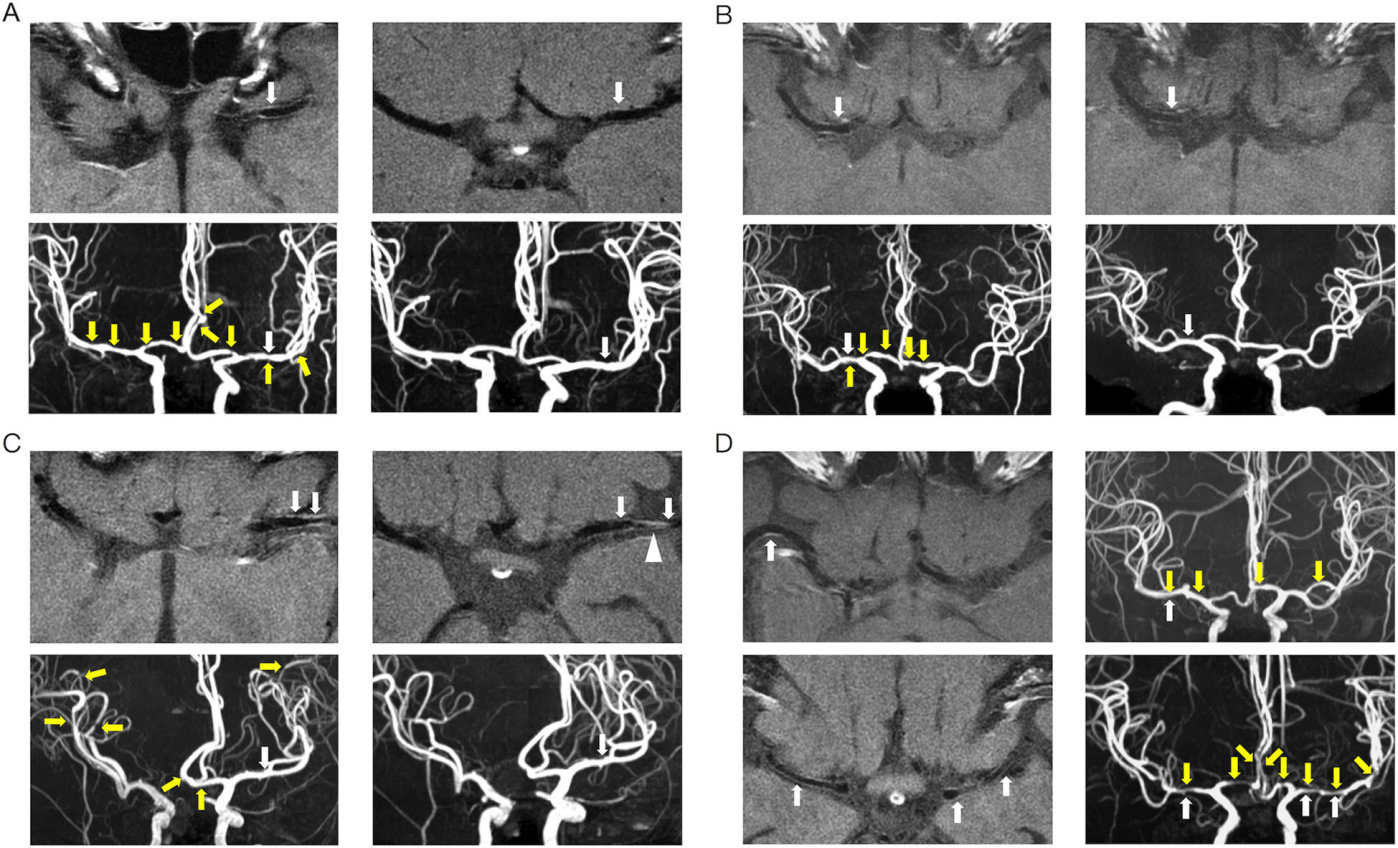
Vascular wall enhancement in patients with RCVS. **a**, initially mild concentric enhancement, vascular imaging obtained 10 days after disease onset in a 48-year-old female; the enhancement was completely resolved at 7 years of follow-up; **b**, initially mild concentric enhancement, vascular imaging obtained 9 days after disease onset in a 52-year-old female; the enhancement was partially resolved 96 days later; **c**, initially marked concentric enhancement, vascular imaging obtained 10 days after disease onset in a 60-year-old female; the enhancement was partially resolved at 4.5 years of follow-up. The white arrowhead in **c** indicates partial volume of vein. Note that the enhanced vascular wall did not concordantly present at the site of vasoconstriction; **d**, upper, initially mild eccentric enhancement, vascular imaging obtained 25 days after disease onset in a 49-year-old female; lower, initially mild concentric enhancement, vascular imaging obtained 15 days after disease onset in a 48-year-old female. White arrows locate vascular wall enhancement. Yellow arrows locate vasoconstriction

Imaging analysis was performed on a radiology information system-picture archiving and communication system. Visual analysis was conducted to evaluate any focal wall thickening and postcontrast enhancement. Postcontrast enhancement was categorized as absent (none or minimal) or present by comparing pre- and post-gadolinium vessel wall imaging; enhancement was considered unequivocal if found in at least two imaging planes. The enhancement was characterized as mild if the arterial wall hyperintensity was mild or patchy (Fig. 1a), and as marked (Fig. 1b) if the arterial wall hyperintensity was strong and diffuse (involving the entire circumference of an arterial segment) in at least two imaging planes. The pattern of enhancement was characterized as concentric if it was uniform and circumferential, and eccentric if nonuniform and noncircumferential [20]. If the patient had mild or marked vascular wall enhancement on the initial scan, they were invited to receive follow-up contrasted T1-FLAIR imaging, independent of their regular MRA follow-up. Any such targeted image findings were independently interpreted by two experienced neuroradiologists (J.F.L. and F.C.C.) who were blinded to the clinical data. The differences in grading were resolved by consensus.

### Transcranial color-coded sonographic studies

Each patients’ transcranial color-coded Doppler sonography was performed on the same day as the corresponding MRA. Mean flow velocities of major cerebral arteries, including the anterior cerebral arteries (ACAs), MCAs, posterior cerebral arteries, and basilar artery were recorded [6]. For bilateral vessels, the averaged velocity of both sides was taken as the mean velocity and maximal velocity was obtained from the side with a greater velocity.

### Clinical follow-up

All eligible patients were followed up until their headaches subsided or the MRA follow-up endpoint. Patients with enhanced vessel walls were invited for an optional follow-up exploratory MRA study. As a result, the follow-up duration was quite variable across patients, but the information obtained may be useful for future follow-up study. The last follow-up was completed in 2017.

### Statistical analysis

Descriptive statistics are presented as means ± standard deviations or percentages. Comparisons between two or more sets of normally distributed data were carried out with the *t-*tests (independent or paired) or one-way analyses of variance (ANOVAs). If normality was not assumed, the differences between two sets of data were tested with the Mann-Whitney U test, and the differences between three sets of data with the Kruskal Wallis test. For correlations between two continuous variables, we calculated the Pearson correlation coefficient, *r*. Predictors of vascular wall enhancement were identified by multiple logistic regression analyses. Statistical significance was set at *p* < 0.05. All analyses were performed with the IBM SPSS Statistics software package, version 18.0.

## Results

### Demographic profile

After excluding patients with poor vascular wall imaging quality or initial brain imaging beyond 30 days, 48 of 62 recruited RCVS patients remained in the final analysis. They were mostly women (42/48; 87.5%) with a mean age of 50.5 ± 9.4 years (range, 27–66 years). Triggers of their thunderclap headaches included defecation (37.5%), bathing (27.1%), intense emotions (20.8%), sex (18.8%), exertion (10.4%), and coughing (6.3%). A total of 12 patients received a spinal tap. All their CSF was clear and colorless, and pressure, cell count and metabolic analyses were within normal limit.

### Ictal-stage vascular wall imaging

The mean latency from presentation to initial vessel wall imaging was 11.9 ± 7.1 days (range 1–30 days). The characteristics of the enhancement are presented in Table 1. A total of 22 patients (45.8%) had enhancement on contrasted vascular wall imaging, including 5 (22.7%) with marked and 17 (77.3%) with mild enhancement. The enhancement was concentric in 16 (72.7%, Fig. 1d, lower) and eccentric in 6 (27.3%, Fig. 1d, upper). The eccentric pattern was present only in vessels with mild enhancement (35.3%). The enhancement was not always co-localized with vasoconstriction. For example, the enhancement was colocalized with vasoconstriction in Fig. 1a, b and d, but incongruous with vasoconstriction in Fig. 1c. The enhancement involved the proximal M1 in 3 (13.6%), distal M1 in 6 (27.3%), and whole M1 in 13 (59.1%). Maximal flow velocity for the MCA and ACA did not differ significantly between patients with vascular wall enhancement (MCA, 114.5 ± 62.7 cm/s; and ACA, 75.1 ± 21.9 cm/s) and those without vascular wall enhancement (MCA, 97.2 ± 29.2 cm/s, *p* = 0.25, independent t test; and ACA, 69.7 ± 18.7 cm/s, *p* = 0.42, independent t test). There was no graded difference when the enhancement was characterized into mild and marked levels (Table 2). The demographics and headache profiles did not significantly differ between the patients with and without vascular wall enhancement (Table 3), except that patients with vascular wall enhancement had less frequent headache attacks (0.6 ± 0.3 per day) and fewer total headache attacks (4.7 ± 4.4) than those without vascular wall enhancement (0.9 ± 1.0 per day, *p* = 0.07, Mann-Whitney U test; 8.4 ± 8.4, *p* = 0.04, Mann-Whitney U test).

**Table 1:** Characteristics of vascular wall enhancement in patients with RCVS

Characteristics of vascular wall enhancement (*n* = 22)	
Segmental location, n (%)	22
Proximal M1	3 (13.6%)
Distal M1	6 (27.3%)
Whole M1 segment	13 (59.1%)
Degree of enhancement, n (%)	22
Mild	17 (77.3%)
Mark	5 (22.7%)
Reversibility, n (%)	14
Complete resolution	5 (35.7%)
Partial resolution	4 (28.6%)
No change	5 (35.7%)
Pattern, n (%)	22
Concentric	16 (72.7%)
Eccentric	6 (27.3%)

**Table 2:** Cerebral blood flow velocity in RCVS patients with different degrees of vascular wall enhancement

	Extent of vessel wall enhancement			
	Absent (*n* = 23)	Mild (*n* = 12)	Marked (*n* = 5)	p
Maximal MCA (cm/s), mean ± SD	97.2 ± 29.2	115.3 ± 69.4	112.4 ± 49.4	0.52
Mean MCA (cm/s), mean ± SD	89.1 ± 23.0	102.6 ± 58.2	103.9 ± 53.3	0.56
Maximal ACA (cm/s), mean ± SD	69.7 ± 18.7	76.0 ± 20.7	73.2 ± 27.2	0.71
Mean ACA (cm/s), mean ± SD	66.2 ± 16.4	68.8 ± 17.8	66.6 ± 21.4	0.92

*ACA* anterior cerebral artery, *MCA* middle cerebral artery, *SEM* standard error of means

**Table 3:** Characteristics of the RCVS patients

	Vascular wall enhancement		p
	Absence (*n* = 26)	Presence (*n* = 22)	
Sex (female), n (%)	23 (88.5%)	19 (86.4%)	1.00
Age (years), man ± SD	49.7 ± 11.1	52.3 ± 7.5	0.53
Time to MRI from onset (days), mean ± SD	11.8 ± 6.9	12.0 ± 7.6	0.93
Vascular risk factors, n (%)			
*Hypertension*	2 (7.7%)	2 (9.1%)	0.86
*Diabetes*	1 (3.8%)	2 (9.1%)	0.59
*CAD*	0 (0.0%)	0 (0.0%)	NA
Headache attacks, n (%)			
*Number of total attacks*	8.4 ± 8.4	4.7 ± 4.4	0.04
*Duration of whole course (d)*	10.5 ± 6.3	8.8 ± 5.1	0.32
*Frequency (number/duration)*	0.9 ± 1.0	0.6 ± 0.3	0.07
Triggers, n (%)			
*Bathing*	8 (30.8%)	5 (22.7%)	0.53
*Exertion*	2 (7.7%)	3 (13.6%)	0.65
*Cough*	2 (7.7%)	1 (4.5%)	1.00
*Defecation*	7 (26.9%)	11 (50.0%)	0.10
*Emotion*	6 (23.1%)	4 (18.2%)	0.68
*Sex*	5 (19.2%)	4 (18.2%)	1.00
Mean number of triggers per patient	1.4 ± 0.9	1.6 ± 1.2	0.65
Mean total WMH volume (ml)	1.1 ± 1.2	1.3 ± 1.8	0.71

*CAD* coronary artery disease, *MRI* magnetic resonance imaging, *SEM* standard error of mean, *WHM* white matter hyperintensity

### Follow-up vascular imaging

Follow-up vascular wall imaging was performed for 12 of the 17 patients with mild enhancement of the vascular wall and 4 of the 5 patients with marked enhancement with a median follow-up interval of 7 months (range, 17 days to 7 years). Analyzable images were obtained in 14 patients, of which 5 (35.7%) showed persistence of the initial enhancement and 9 (64.3%) showed partial or complete resolution of the initial enhancement (Fig. 1). Among 3 patients who received analyzable follow-up vascular wall imaging within 3 months, 1 patient (33%) showed persistence of the initial enhancement and 2 patients (67%) showed a reduction. Among 8 patients who received analyzable follow-up imaging within 3 years, 5 patients (62.5%) had persistent enhancement and 3 patients (37.5%) had a reduction. In patients with initially mild enhancement, the follow-up imaging showed no change in enhancement degree in 4 patients (with 35-, 96-, 168- and 641-day intervals), complete resolution in 4 patients, and partial improvement in 2 patients. Among patients with marked enhancement initially, follow-up vascular imaging showed complete regression in 1 patient after 46 days and residual mild enhancement in 2 patients. One patient had persistently marked enhancement, but the follow-up interval was short (17 days).

## Discussion

In the present study, almost half of the RCVS patients showed some degree of enhancement in contrasted T1-FLAIR vascular imaging. In three fourths of the cases, the vascular wall enhancement was mild, and in the remaining fourth the enhancement was marked. The intensity of enhancement was not associated with MCA or ACA flow velocity. The enhancement of the vascular walls persisted at follow-up in a third of these patients, with a median follow-up duration of 7 months. The proportion of RCVS patients with vascular wall enhancement observed in this cohort was higher than that reported in previous studies [19, 20] and was persistent in some cases, suggesting that vascular wall enhancement may not be a reliable imaging sign as previously thought for clinical differentiation of RCVS from vasculopathy with an inflammatory component.

Differentiation from CNS vasculitis precludes unnecessary invasive brain biopsy, cerebral angiography, and lifelong immunosuppression in RCVS patients [12]. Previous studies with small numbers of patients found that arterial wall enhancement was mild (if present) in a minority of RCVS patients [20], as opposed to the strong wall enhancement frequently observed in vasculitis patients. In our present study, most of the vascular wall enhancement was also mild in the RCVS patients. However, the proportion of patients in which vascular wall enhancement was found was much higher than previously reported (47% vs. 31%) [20], and a fourth of the patients had strong vascular wall enhancement. Notwithstanding, the clinical hallmarks of recurrent thunderclap headaches and the reversibility of vasoconstriction without immunosuppressants in our patients support their being diagnosed with RCVS over CNS vasculitis.

Arterial wall enhancement in contrasted vascular imaging may reveal an inflammatory component of RCVS pathology. Although inflammation is not considered to play a key role in the pathogenesis of RCVS, prolonged vasoconstriction per se has been proposed to be associated with an inflammatory process [23, 27]. Of note, an inflammatory cascade has been reported in cerebral vasospasm in SAH [28]. Although the pathologies of RCVS and SAH would be expected to differ from each other, they might share some pathomechanisms. For example, oxidative stress and endothelial dysfunction, which contribute to the vascular wall inflammation, have also been noted in patients with RCVS [29, 30]. Additionally, a postulated mechanism of cocaine-induced vasculitis includes cerebrovascular smooth muscle cells apoptosis and promotion of leukocyte migration across cerebral vascular walls [23, 31, 32], producing vessel wall inflammation. Because cocaine-induced vasculitis is considered a spectral disorder of RCVS [9, 12, 24], it is reasonable to deduce that vascular wall inflammation exists in at least some patients with secondary RCVS. Prolonged vasoconstriction has been hypothesized to contribute to the development of secondary angiitis [27]; similar mechanisms might also contribute to prolonged vascular wall enhancement. However, these were purely speculative; the nature of the persistent/residual vascular wall enhancement remains to be elucidated.

The enhancement of diseased vessels in RCVS was suggested to be reversible in a study completed in the USA [20]. In that study, 8 out of 9 RCVS patients showed complete resolution of their initial vascular wall imaging findings, with only 1 having minimal residual wall thickening after a median follow-up period of 3.5 months [20]. In contrast, one third of our patients with follow-up vascular imaging had persistent mild enhancement after a median period of 3 months (longest period, 21 months). Even among the 10 patients with some level of reduced enhancement on follow-up imaging (follow-up range, 55–95 months), four had residual enhancement. Slower resolution or greater persistence of the enhancement has been observed in atherosclerosis of the intracranial vessels [33], particularly if the enhancement is eccentric and heterogeneous with mild to moderate intensity [13]. Although atherosclerosis risk factors were not commonly present in our patients with persistent or residual enhancement (one patient had hypertension, and one patient had hypertension and diabetes), we could not completely exclude the possibility of subclinical atherosclerosis in our patients. Particularly, it has been found that subclinical atherosclerosis can be present in as high as 50% of patients with low cardiovascular risk [34] and about 60% of asymptomatic patients [34, 35], and that intracranial atherosclerosis is more prevalent in Asians [36]. Compared with the study by Mossa-Basha et al. [37], we focused more on the reversibility of vascular wall enhancement in RCVS, finding that the vascular wall enhancement was not always reversible in patients with RCVS, probably due to etiological heterogeneity. Hence, although vascular wall imaging is a powerful and reliable tool for evaluating diseases involving intracranial vessels, the use of it as an ancillary diagnostic tool for RCVS required deliberation. The persistence of vascular wall enhancement in RCVS did not depreciate the value of vessel wall imaging for differentiation of nonocclusive intracranial vasculopathies, but instead reminded the clinicians not making the diagnosis solely based on reversibility of the enhancement.

The headache characteristics of RCVS [38] are distinct from the primary headaches such as migraine [39] or cluster headache [40]. Although 20% of the patients with RCVS have pre-existing migraine [38], the cardiovascular or neurological comorbidities known to be associated with migraine [41–43] have not been well explored in patients with RCVS. Because both migraine [44] and RCVS [45] are associated increased risks of white matter hyperintensities, there could be some shared mechanisms between these two disorders. A higher headache frequency and long-term migraine may worsen the cardio-metabolic profile in migraineurs [44], which might partially be mediated by circulating microRNAs associated with vascular function [46, 47]. Whether similar mechanisms could contribute to RCVS pathogenesis or the imaging findings disclosed in this study deserve further investigation.

The present study had several limitations. First, we did not include a control group because doing so would involve unnecessary exposure of subjects to the potential risks of gadolinium deposition in the brain [48]. Second, because we did not recruit patients with secondary causes of RCVS, one should be cautious to extrapolate the findings to the general pathogenesis of RCVS. Given that secondary causes of RCVS are far less common than idiopathic ones in Asian patients [38, 49], elucidating the pathogenesis of the latter was our major concern. Third, although follow-up MRA evaluations for confirming vasoconstriction reversibility were obtained for all of our patients, the retention rate for vascular imaging was 70.8%, mainly due to the undesirable requirement of contrast injection. Fourth, the magnitudes of vascular wall enhancement observed in the present study do not correspond precisely with severity levels defined in the previous reports. However, vascular wall enhancement level differences across studies may reflect the particular machines, settings, and protocols used. In our study, we focused more on the presence of vascular wall enhancement, and the temporal change of the enhancement, both may have little to do with the degrees of the initial vascular wall enhancement. Fifth, these patients received the same treatment (nimodipine) but the resolution of enhancement was heterogeneous, so we cannot be sure if that enhancement of vascular wall imaging is altered by medical treatment. Sixth, the reluctance of patients to undergo spinal tap in Taiwanese society precluded CSF studies in many patients; therefore, the differential diagnosis of RCVS in our practice heavily relied on the presence of the clinical hallmark of RCVS (i.e. recurrent thunderclap headaches) and imaging findings (to demonstrate the reversibility of vasoconstrictions and to exclude SAH or other secondary causes of thunderclap headaches by susceptibility weighted imaging or other MR sequences [38, 45]. Nevertheless, the characteristics of the patients who received spinal tap were not different from those who did not receive spinal tap. Finally, our study could not confirm how long the persistent or residual enhancement could last. Studies with a longer follow-up period are needed.

## Conclusion

Demographics, clinical profiles, and cerebral artery flow velocities were similar across patients with versus without vascular wall enhancement. Half of the RCVS patients had enhancement of diseased vessels and it was persistent for one third of them, so vascular wall enhancement may not be a reliable imaging marker for differentiating RCVS from central nervous system vasculitis or subclinical atherosclerosis. The clinical implication of our findings is that the differentiation of RCVS from other intracranial vasculopathy should not be made solely based on vascular wall imaging.

## Abbreviations

ACA: Anterior cerebral artery
ANOVA: Analyses of variance
CNS: Central nervous system
FLAIR: Fluid-attenuated inversion recovery
ICHD-2: International Classification of Headache Disorders, second edition
MCA: Middle cerebral artery
MRA: Magnetic resonance angiogram
MRI: Magnetic resonance imaging
RCVS: Reversible cerebral vasoconstriction syndrome
SAH: Subarachnoid hemorrhage
